# An antagonist of the retinoid X receptor reduces the viability of *Trichuris muris in vitro*

**DOI:** 10.1186/1471-2334-14-520

**Published:** 2014-09-27

**Authors:** Rebecca JM Hurst, Thomas Hopwood, Amanda L Gallagher, Frederick A Partridge, Timothy Burgis, David B Sattelle, Kathryn J Else

**Affiliations:** Faculty of Life Sciences, University of Manchester, AV Hill Building, Oxford Road, Manchester, M13 9PT UK; Wolfson Institute for Biomedical Research, Division of Medicine, University College London, Cruciform Building, Gower Street, London, WC1E 6BT UK

**Keywords:** RXR, HX531, *Trichuris muris*, Nematode, Viability, Anthelmintic

## Abstract

**Background:**

Trichuriasis is a parasitic disease caused by the human whipworm, *Trichuris trichiura*. It affects millions worldwide, particularly in the tropics. This nematode parasite burrows into the colonic epithelium resulting in inflammation and morbidity, especially in children. Current treatment relies mainly on general anthelmintics such as mebendazole but resistance to these drugs is increasingly problematic. Therefore, new treatments are urgently required.

**Methods:**

The prospect of using the retinoid X receptor (RXR) antagonist HX531 as a novel anthelmintic was investigated by carrying out multiple viability assays with the mouse whipworm *Trichuris muris*.

**Results:**

HX531 reduced both the motility and viability of *T. muris* at its L3, L4 and adult stages. Further, bioinformatic analyses show that the *T. muris* genome possesses an RXR-like receptor, a possible target for HX531.

**Conclusions:**

The study suggested that Trichuris-specific RXR antagonists may be a source of much-needed novel anthelmintic candidates for the treatment of trichuriasis. The identification of an RXR-like sequence in the *T. muris* genome also paves the way for further research based on this new anthelmintic lead compound.

**Electronic supplementary material:**

The online version of this article (doi:10.1186/1471-2334-14-520) contains supplementary material, which is available to authorized users.

## Background

*Trichuris trichiura*, the human whipworm, is an intestinal nematode affecting over 450 million people worldwide, particularly those living in the tropics [1]. *T. trichiura* burrows into the epithelial lining of the colon to cause inflammation, and in cases of high parasite burden can lead to diarrhoea, poor growth, finger clubbing, anaemia and rectal prolapse [2]. Other members of the *Trichuris* genus can have damaging effects on livestock, decreasing productivity and thus causing farming communities to be trapped in a state of poverty. Currently, the only treatment for trichuriasis is the distribution of anthelmintics, such as mebendazole. However, these drugs only affect the adult stage of the parasite, and resistance is becoming increasingly common; in some cases mebendazole has been reported to be as low as 45% effective [3]. Even when parasites are cleared from the host, re-infection from the environment is common. Therefore, there is an urgent medical need for new treatments for this parasitic infection.

Nuclear hormone receptors such as the retinoid X receptor (RXR) play many vital roles in the development of organisms [4]. Indeed, RXRα KO mice are not viable [5]. In mammals, there are three isoforms of intracellular RXR receptors (α, β and γ), and these function as hetero- or homo-dimers [6]. Upon dimerisation, co-activator or co-repressor molecules are recruited and the transcription of genes under the control of these receptors is modified. The natural ligand for RXR homodimers is 9-cis-retinoic acid, however, since RXR can heterodimerise with other nuclear hormone receptors such as the retinoic acid receptor (RAR), peroxisome proliferator-activated receptor (PPAR) and vitamin D receptor (VDR), its ligands also include all-trans-retinoic acid, fatty acids and vitamin D [7, 8].

Since RXR receptors are highly evolutionarily conserved [9], it is not unreasonable to propose that parasites may also have similar receptors. Indeed, the parasitic trematode *Schistosoma mansoni* has been shown to possess RXR-like receptors [10]. In this parasite, a role for such receptors in the expression of female genes has been proposed [10]. Retinoic acid may have several important functions in parasites. For example, *Onchocerca volvulus* secretes a protein which sequesters retinoic acid [11]. Also, the *Ascaris suum* ABA-1 allergen has demonstrated retinol and retinoic acid binding capabilities [12] and *Brugia malayi* has been shown to take up radio-labelled retinoic acid [13]. Retinoic acid is a ligand of RXR, therefore this may suggest important roles of RXR in parasitic species. This study tested whether RXR is important in the biology of *Trichuris muris*, the laboratory model parasite for human trichuriasis, by investigating the actions of an RXR-active compound on *T. muris* viability. *T. muris* is maintained in laboratory mice by oral infection of embryonated eggs. Eggs hatch in the large intestine and progress through four larval stages before becoming fecund adults at around day 35 post-infection [14].

Bioinformatic analysis of the *T. muris* genome [15] demonstrated that *T. muris* possesses an RXR-like receptor. Two synthetic retinoids known to modulate the function of mammalian RXR were used to target worm viability *in vitro*. HX630 is an RXR agonist which can bind to RXR and also to nuclear hormone heterodimers, which include RXR as a partner, thereby activating RXR signalling, including RXR-RAR signalling [16, 17]. Conversely, HX531 is an antagonist of RXR and has been shown to inhibit both RXR-RAR heterodimers and PPARγ-RXR heterodimers in mammalian cells [18, 19]. To our knowledge, neither HX630 nor HX531 have been tested for anti-parasitic activity. The blocking of the RXR receptor by HX531 reported here results in reduced viability of the *T. muris* parasite at L3, L4 and adult stages of its lifecycle. Three different assays were used to assess worm viability (motility scoring, MTT assay and a novel camera-based method) and their utility as a measure of worm health was compared. The findings suggest that RXR may represent a much-needed, novel drug target for the treatment of trichuriasis.

## Methods

### Animals, parasites and compounds

Immunodeficient SCID mice were bred by the Biological Services Facility (BSF, University of Manchester, UK). All procedures carried out on animals were performed under a Home Office licence approved by the University of Manchester Ethical Review body, and complied, at all times, with UK laws and regulations. Mice were housed in sterile conditions and all experiments were carried out in accordance with the UK Animals (Scientific Procedures) Act 1986.

The *T. muris* (Edinburgh (E) strain) was maintained as previously described by Wakelin [20]. For *in vitro* assays, SCID mice were infected with approximately 200 infective *T. muris* eggs by oral gavage. At days 24, 31 and 40, mice were sacrificed and the caecum and colon were removed, opened longitudinally and washed in pre-warmed 0.9% NaCl. Guts were incubated in 0.9% NaCl at 37°C for 1 h. Parasites were isolated and 4 parasites per well were transferred into a 96-well plate containing 100 μl fresh RPMI 1640 medium supplemented with penicillin (500U/ml) and streptomycin (500 μg/ml). Compounds of interest were added to make a final volume of 200 μl per well. Final concentrations of the compounds were as follows: Mebendazole (Ovex) (McNeil, Berkshire, UK) 200, 100 and 50 μg/ml; HX630 (RXR Agonist) and HX531 (RXR Antagonist) (both kind gifts from Hiroykui Kagechika, Tokyo Medical and Dental University, Japan) 100, 10 and 1 μM. HX630 and HX531 were dissolved in 0.2% DMSO, and therefore the vehicle treatment consisted of 0.2% DMSO without any active drug. Bleach (5%) was used as a positive control. Negative control wells contained 200 μl RPMI 1640 media plus penicillin (500U/ml) and streptomycin (500 μg/ml).

### Motility assay

Following incubation with test compounds for 24 h, the motility of *T. muris* larvae was assessed using an Olympus SD-ILK microscope. A scale of 0–3 was used; 0 = dead, 1 = very low motility, 2 = low motility and 3 = normal motility [21].

### MTT assay

After 24 h incubation with the test compounds, worms were transferred into 100 μl fresh RPMI 1640 medium plus penicillin (500U/ml) and streptomycin (500 μg/ml). 100 μl MTT reagent (Thiazolyl blue tetrazolium bromide; Sigma, Dorset, UK) was added to each well at a concentration of 6 mg/ml in deionised water. The plate was then incubated at 37°C, 5% CO_2_ for 1 h. The larvae were transferred into a new 96 well plate containing 200 μl DMSO and again incubated at 37°C, 5% CO_2_ for 1 h to solubilise the formazan crystals. The worms were removed and absorbance of formazan was measured at a wavelength of 490 nm using a microplate reader (Dynex Technologies, VA, USA).

### Camera motility assay

Parasites were filmed recorded in 96 well plates for 200 frames at 10 frames per second using an Andor Neo camera and LED array illumination. Parasite motility was determined by an algorithm that thresholds the movie based on variance in pixel intensity over time and then counts motile pixels.

### Bioinformatics

Protein sequences from the *T. muris* gene set 2.1 (Feb 2013) were obtained from http://www.ftp.sanger.ac.uk/pub/pathogens/Trichuris/muris/ and used to build a local BLAST database. The human RXR alpha protein sequence was queried against this database using blastp 2.2.29+. The most significant four matches were aligned, using Clustal Omega, against representatives of the RXR gene family and the related NR2F gene family, and selected nematode genes annotated as RXR homologues. The more distantly related *C. elegans* protein NHR-49 was used as an out group. A phylogenetic tree was constructed using the neighbour-joining method.

### Statistics

Statistical analyses were performed using the unpaired t-test or one way ANOVA (with Tukey’s multiple comparison post-hoc test), as appropriate, with the statistical package GraphPad Prism (Version 6). A p value of less than 0.05 was considered significant.

## Results

### The *T. muris*genome contains an RXRα-like sequence

**Figure 1:**
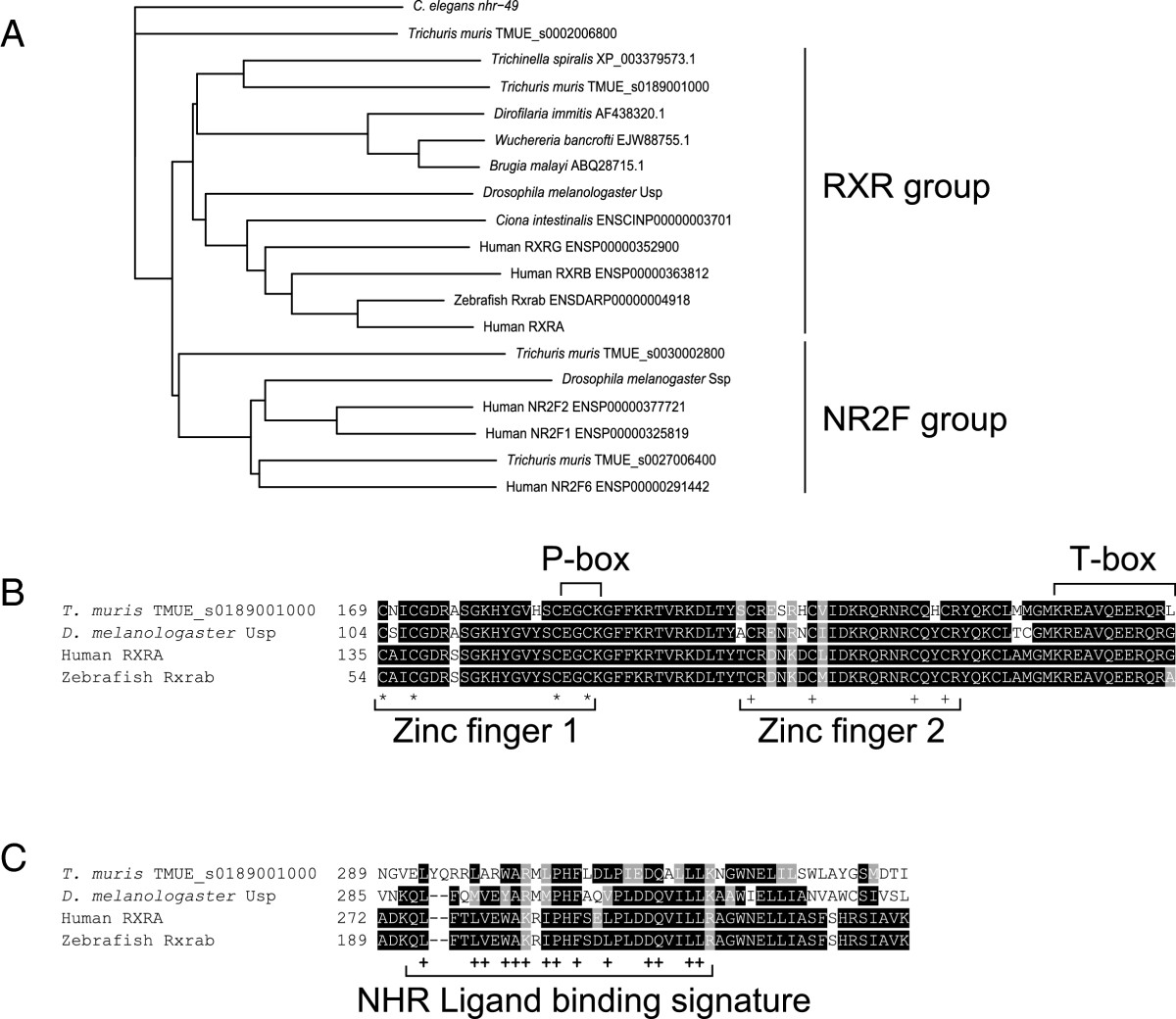
**Neighbour-joining tree of** ***T*** **.** ***muris*** **homologues of RXR proteins. (A)** The human RXRα protein sequence was queried against the *T. muris* gene set 2.1 (Feb 2013). The most significant four matches were aligned against representatives of the RXR gene family, (including sequences from other nematodes annotated as RXR homologues) and representatives of the related NR2F gene family. The more distantly related *C. elegans* protein NHR-49 was used as an outgroup. The phylogenetic tree shows that *T. muris* has a single orthologue of the RXR proteins, TMUE_s0189001000. **(B)** Alignment showing conservation of the DNA-binding domain. **(C)** Alignment showing conservation within the ligand-binding domain.

Given the availability of the *T. muris* genome, the conserved nature of the RXR nuclear hormone receptor and the knowledge that other parasitic nematodes have retinoic acid binding capacity, the human RXRα protein sequence was queried against the *T. muris* gene set 2.1 (Feb 2013). The most significant four matches were aligned against representatives of the RXR gene family, including genes from other nematodes annotated as RXR homologues [22], and representatives of the related NR2F gene family. The more distantly related *C. elegans* protein NHR-49 was used as an outgroup. Figure 1A is a neighbour joining tree showing the relationship between these sequences. *T. muris* appears to have a single orthologue of the RXR genes [TMUE:s0189001000], which encodes a protein that is 55% identical to human RXRα.

To support the hypothesis that this gene encodes a functional receptor, sequence conservation was analysed more closely. Key residues in the DNA-binding domain, including both zinc fingers and the P-box and T-box sequences [23] are conserved between human RXR and *T. muris* sequences (Figure 1B). Residues in the ligand-binding domain known to be important for functionality of nuclear hormone receptors [24] are also conserved (Figure 1C). Whilst this does not demonstrate that *T. muris* TMUE_s0189001000 acts functionally as a retinoid X receptor, is does suggest that it is a functional nuclear hormone receptor and is closely related to the RXR family. Furthermore, homologues of this receptor are found in other parasitic nematodes (Figure 1A), suggesting RXR-active compounds have the potential to interfere with parasite biology.

### The RXR antagonist HX531 reduced the viability of L3/L4 *T. muris in vitro*

**Figure 2:**
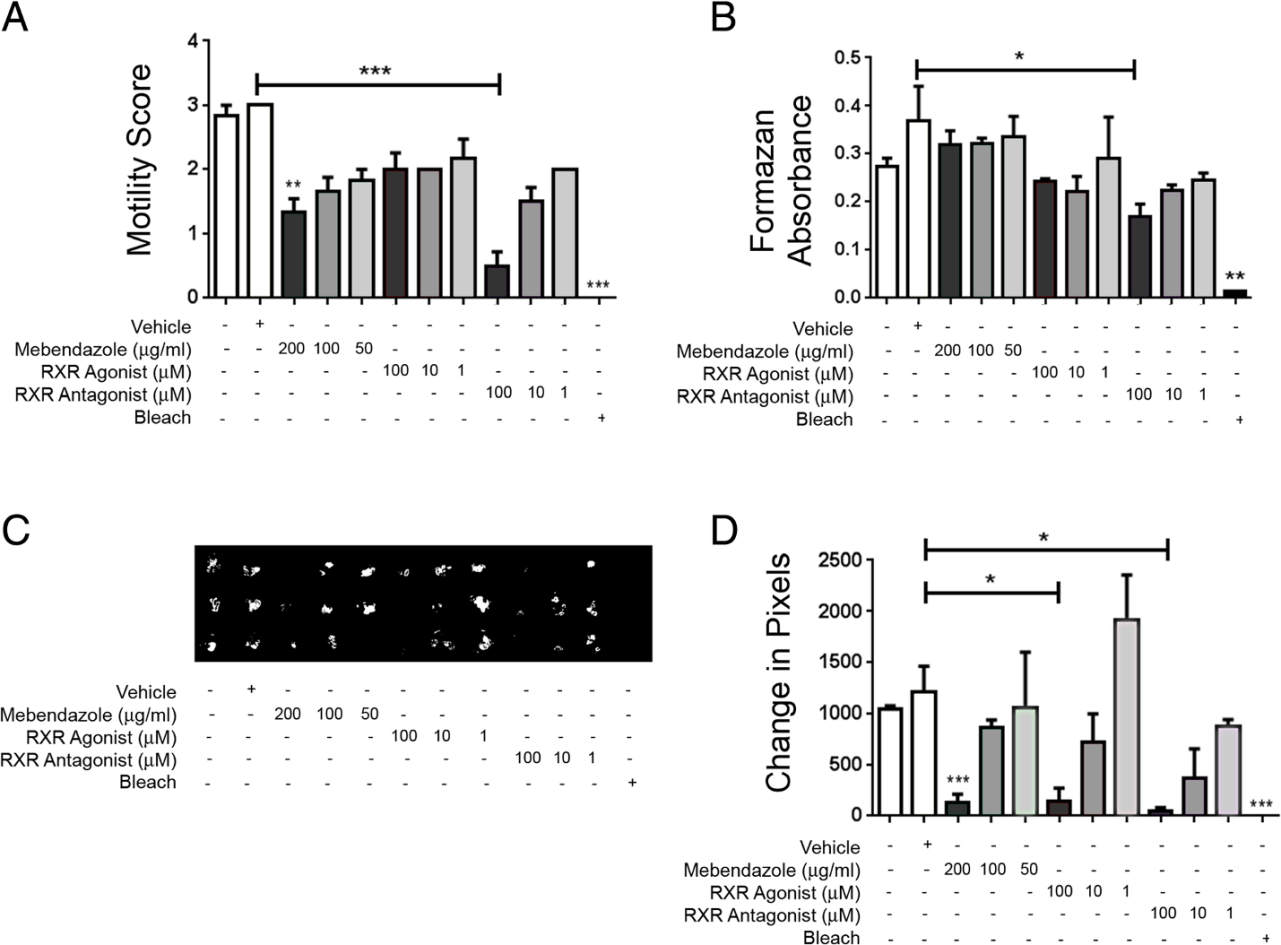
**The RXR antagonist HX531 reduced the viability of L3/L4** ***T. muris in vitro*** **.** Worms were extracted from mice at day 24 post-infection and incubated for 24 hours with vehicle, the anthelmintic mebendazole, the RXR agonist HX630, the RXR antagonist HX531 or bleach. Worm viability was assessed using **(A)** a motility score, in which 0 = dead, 1 = very low motility, 2 = low motility and 3 = normal motility, or **(B)** MTT assay. An Andor camera was used to capture the movement of *T. muris* by filming for 20s. Pixel changes from frame to frame were captured as an indicator or motility **(C)** and quantified using Matlab software **(D)**. Data are representative of two independent experiments (n = 3). *p < 0.05, **p < 0.01, ***p < 0.001, compared to controls, unless denoted otherwise.

To assess whether the RXR antagonist HX531 affects the viability of the L3 and L4 stages of the *T. muris* parasite, larvae were extracted from infected mice at day 24 post infection, a time point when both L3 and early-moulted L4 larvae are present. Larvae were incubated with HX531 at concentrations of 100, 10 and 1 μM for 24 h. As controls, parasites were also incubated with the anthelmintic mebendazole (200, 100 and 50 μg/ml), RXR agonist (HX630; 100, 10 and 1 μM), vehicle, media only or bleach (1:100). When incubated with mebendazole (200 μg/ml) or bleach, both larval stages had a significantly reduced motility score compared to media-only controls (*p* < 0.01 and *p* < 0.001, respectively) (Figure 2A). Interestingly, HX531 (100 μM)-treated larvae also had a significantly reduced motility score compared to vehicle treatment (*p* < 0.001) (Figure 2A), as well as reduced viability shown by the MTT assay data (*p* < 0.05) (Figure 2B). Worm motility was also assessed using a camera which tracked the movement of the worm followed by measuring changing pixels in a frame-by-frame analysis. Using this assay, L3-L4 *T. muris* larvae incubated with mebendazole (200 μg/ml) or bleach again had significantly reduced motility compared to media-only controls (*p* < 0.001 for both) (Figure 2C and D). However, both the RXR antagonist (100 μM)-treated larvae and RXR agonist (100 μM)-treated larvae showed a significantly reduced motility compared to vehicle treatment (*p* < 0.05 for both) (Figure 2C and D).

### The RXR antagonist HX531 reduced the viability of L4 and adult *T. muris*, *in vitro*

**Figure 3:**
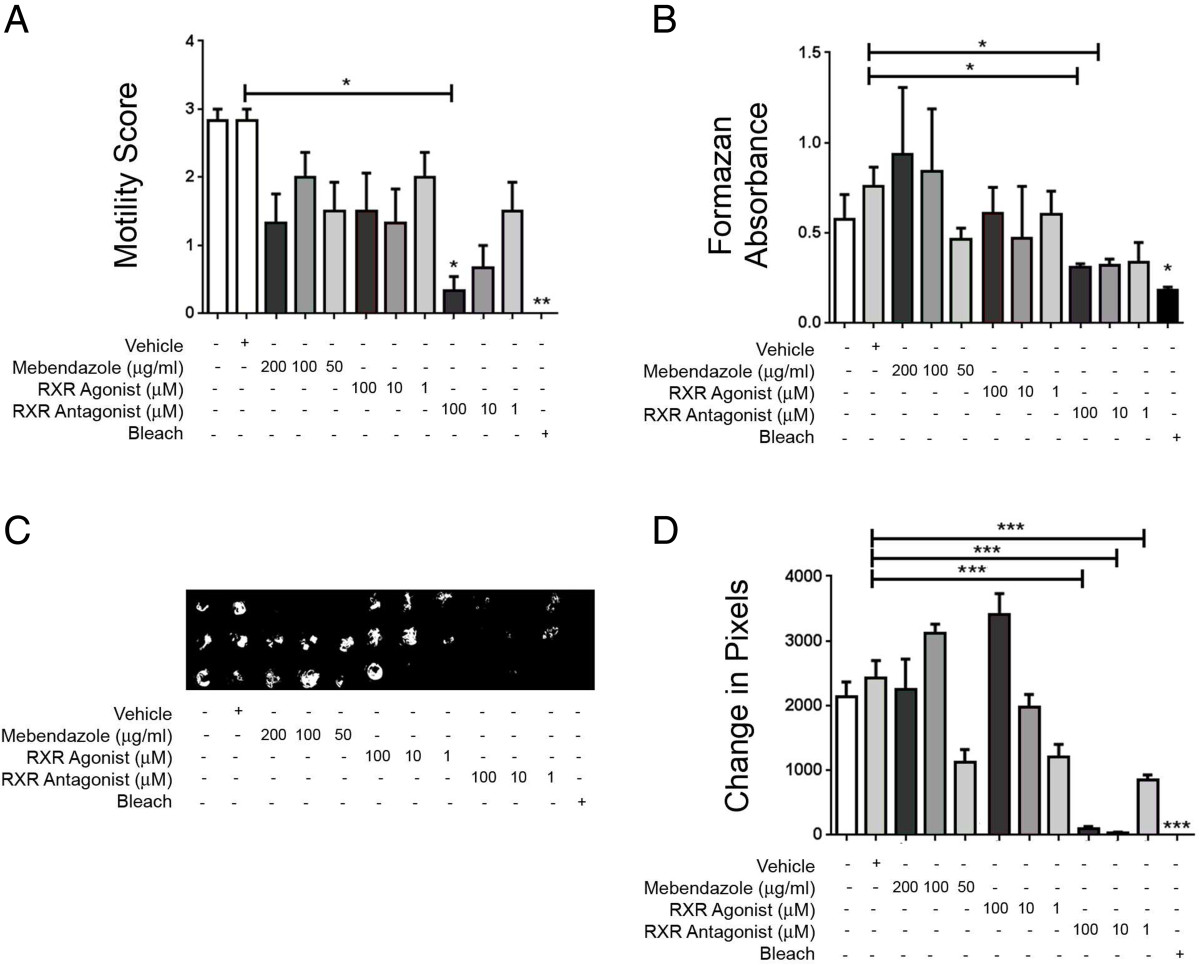
**The RXR antagonist HX531 reduced the viability of L4/adult** ***T. muris in vitro*** **.** Worms were extracted from mice at day 31 post-infection and incubated for 24 hours with vehicle, the anthelmintics mebendazole and pyrantel embonate, the RXR agonist HX630, the RXR antagonist HX531 or bleach. Worm viability was assessed using **(A)** a motility score, in which 0 = dead, 1 = very low motility, 2 = low motility and 3 = normal motility, or **(B)** MTT assay. An Andor camera was used to capture the movement of *T. muris* by filming for 20s. Pixel changes from frame to frame were captured as an indicator or motility **(C)** and quantified using Matlab software **(D)**. Data are representative of two independent experiments. (n = 3). *p < 0.05, **p < 0.01, ***p < 0.001, compared to controls, unless denoted otherwise.

L4-adult *T. muris* were recovered at day 31 post-infection, a time point when both newly moulted adults are present but some worms still remain as L4. Incubation with bleach significantly reduced motility scores compared to media-only controls (p < 0.01 for both) (Figure 3A). Again, RXR antagonist treatment (100 μM) resulted in significantly reduced motility scores compared to vehicle controls (*p* < 0.001) (Figure 3A). Using the motility score assay, neither mebendazole nor the RXR agonist had any effect on parasite viability (Figure 3A). MTT assay data showed that RXR antagonist treatment at both 100 μM and 10 μM significantly reduced viability compared to vehicle-treated controls (*p* < 0.05 for both) (Figure 3B). None of the other treatments had any significant effect on parasite viability compared to controls in the MTT assay (Figure 3B). Camera analysis of worm movement showed a reduced motility in parasites incubated with bleach, compared to media only controls (*p* < 0.001 for all) (Figure 3C and D). RXR antagonist treatment also caused a significant reduction in movement compared to vehicle controls at all three concentrations tested (100, 10 and 1 μM) (p < 0.001 for all) (Figure 3C and D). Mebendazole and RXR agonist-treatment had no significant effect on worm motility using this analysis (Figure 3C and D).

### The RXR antagonist HX531 reduced the viability of adult *T. muris*, *in vitro*

**Figure 4:**
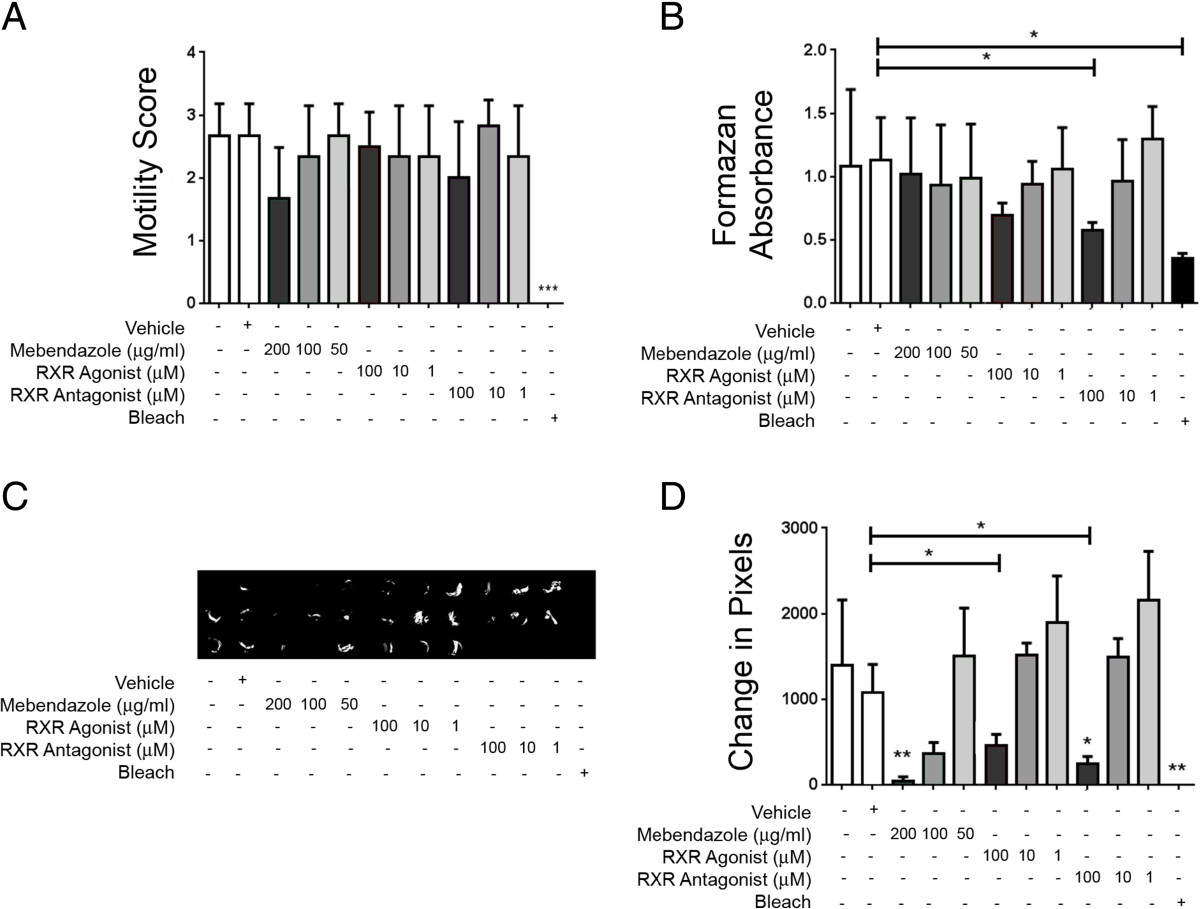
**The RXR antagonist HX531 reduced the viability of adult** ***T. muris in vitro*** **.** Worms were extracted from mice at day 40 post-infection and incubated for 24 h with vehicle, the anthelmintics mebendazole and pyrantel embonate, the RXR agonist HX630, the RXR antagonist HX531 or bleach. Worm viability was assessed using **(A)** a motility score, in which 0 = dead, 1 = very low motility, 2 = low motility and 3 = normal motility, or **(B)** MTT assay. An Andor camera was used to capture the movement of *T. muris* by filming for 20s. Pixel changes from frame to frame were captured as an indicator or motility **(C)** and quantified using Matlab software **(D)**. Data are representative of two independent experiments. (n = 3). *p < 0.05, **p < 0.01, ***p < 0.001, compared to controls, unless denoted otherwise.

To confirm the effects of RXR compounds on adult *T. muris* alone, worms were extracted at day 40 post-infection a time at which all worms present are mature adults. The adult parasites had a reduced motility score when incubated with bleach compared to media only controls (*p* < 0.001) (Figure 4A). None of the other treatments had a significant effect on *T. muris* motility scores (Figure 4A). However, the MTT assay revealed a decreased parasite viability with RXR antagonist treatment (100 μM), as well as the bleach, when compared to vehicle controls (*p* < 0.05 for both) (Figure 4B). Mebendazole or RXR agonist treatment showed no significant impact on worm vitality at any of the concentrations tested, using the MTT assay (Figure 4B). However, the camera-based analysis of worm movement showed that mebendazole (200 μg/ml) as well as bleach reduced worm motility compared to media only controls (*p* < 0.01 for both) (Figure 4C and D). RXR antagonist (100 μM) treatment also caused a significant attenuation of worm motility compared to vehicle controls (*p* < 0.05), although this was also observed with RXR agonist (100 μM) treatment (p < 0.05) (Figure 4C and D).

Overall, the RXR antagonist HX531 emerges as a candidate lead compound for a novel anthelmintic, acting on both larval and adult stages of the parasite. Its anti-parasitic activity was detected using three different vitality assays for worms recovered at day 21 (L3/L4) and day 31 (L4/adult) and two out of three assays for the day 40 mature adult stage parasites.

## Discussion

Gastrointestinal helminth infections affect over 1 billion people worldwide and are a significant cause of human and livestock morbidity. With resistance to current treatments becoming increasingly problematic and with the lack of any vaccine, novel anthelmintics are urgently required [3]. In this study, it was demonstrated for the first time that RXR antagonists show anthelmintic activity and may therefore serve as important lead compounds in the search for new treatments for nematode infections. At L3, L4 and adult stages of the parasite, the human RXR antagonist HX531 was able to reduce both the motility and viability of *T. muris* larvae *in vitro*, as determined by a range of viability assays. The RXR agonist HX630 also reduced worm motility in the L3/L4 and mature adult only stages, however this was only observed in one viability assay. Earlier work has demonstrated that synthetic retinoids that are weak RAR agonists are able to interfere with the development of filarial nematodes *in vitro*[25, 26]. Our studies of an RXR agonist and antagonist in three different parasite viability assays, show that the RXR antagonist (HX531) displays the most robust anthelmintic properties.

RXR is a nuclear hormone receptor which can heterodimerise with many different partners, such as the vitamin D receptor (VDR) or retinoic acid receptor (RAR). Owing to its promiscuous nature, RXR is responsible for controlling the transcription of a variety of different genes and therefore has a wide range of physiological effects, including those essential for homeostasis and development. For example, the human RXR controls fundamental biological processes from reproduction to embryonic development to cell proliferation [8, 27]. Thus, it is not surprising that disruption of the RXR signalling pathway could have a detrimental effect on organism viability. An RXR-like receptor has been shown to exist in the trematode parasite *Schistosoma mansoni*. Studies have shown that the SmRXR is expressed constitutively and therefore may have many roles throughout the parasite’s lifecycle [28]. The findings in this investigation are in accord with this, as RXR antagonism was able to reduce *T. muris* viability at three different lifecycle stages; L3, L4 and adults.

The study demonstrated, for the first time, that *T. muris* has an RXR-like sequence in its genome. This is important not only in terms of novel drug targets, but also in the context of helminth biology. The presence of this receptor indicated that ligands for RXR may be important in *Trichuris* health. Current understanding of *T. muris* feeding processes are limited. It is thought that the stichosome gland cells release enzymes which break-down food externally and then nutrients are taken up by bacillary cells [29, 30]. If RXR is important for *T. muris* health, these nutrients might include those that act through RXR signalling, such as retinoic acid or vitamin D.

The use of vitamin A to treat nematode infections is controversial. A study has shown that vitamin A supplementation to *Ascaris*-infected individuals can promote worm-clearing Th2 responses [31]. In contrast, we have previously reported that activation of the vitamin A receptor, RAR, can cause increased inflammation in the host during *T. muris* infection [32]. Remarkably, vitamin A deficiency is associated with an increased ability to control *T. muris* infections via innate lymphoid cell type 2 dependent immunity [33]. The data presented in the current paper suggest that exploring the impact of vitamin A or its metabolite retinoic acid on the nematode itself as well as on its host responses is of considerable interest.

This study showed that the half-maximal inhibitory concentration for HX531 on *T. muris* viability was between 10 μM and 100 μM. This was higher than 0.29 μM- the reported IC50 of HX531 in mammalian kidney cells [34]. This difference in potency may reflect the variations in RXR sequence between mammals and parasites. Bioinformatic analysis of the *T. muris* genome revealed that this parasite has a sequence with 55% homology with the human RXRα nuclear hormone receptor. Ideally anti-parasitic drugs should affect the parasite and not the host. For example, mebendazole binds to nematode β-tubulin to prevent transport of secretory vesicles [35]. However, a current, widely-used anthelmintic, ivermectin, has recently been identified as a novel FXR ligand [36] in additions to its actions on glutamate-gated chloride channels [37]. FXR is a nuclear hormone receptor which heterodimerises with RXR and thus this supports the principle of targeting nuclear hormone receptors in the development of novel anthelmintics. Looking to the future, the development of a novel parasite-specific RXR antagonist remains a preferable solution.

A number of nematode viability assays exist [21]. This study used a novel technique for measuring nematode vitality. An Andor camera was employed to video parasite movement over a 10–20 s period, and Matlab software was used to calculate the change in pixels resulting from worm movement. Overall, this method gave results consistent with the other viability assays, but provided several advantages over other methods. This assay was completely objective and did not require blinding. Results were reproducible between replicates and the speed of this system advocates its use for high-throughput screening of test compounds to determine their anthelmintic capacity. However, the camera motility assay also revealed effects on *T. muris* after incubation with the RXR agonist. Thus, this highlights the necessity of carrying out multiple assays when testing drug effects on parasite vitality. The techniques used here in showing an RXR antagonist to be a candidate anthelmintic lead compound may gain further traction as the *T. muris* genome [15] highlights additional, druggable targets to pursue.

## Conclusion

In conclusion, RXR antagonism may be a useful novel method for treating trichuriasis, by reducing worm vitality at different lifecycle stages. This study also showed, that the *T. muris* genome has an RXR-like sequence, which may open new doors in the understanding of *Trichuris* biology.

## Authors’ original submitted files for images

Below are the links to the authors’ original submitted files for images. Authors’ original file for figure 1 Authors’ original file for figure 2 Authors’ original file for figure 3 Authors’ original file for figure 4
