# Supplementary figures and images for: An antagonist of the retinoid X receptor reduces the viability of Trichuris muris in vitro
Source: BMC Infect Dis. 2014 Sep 27;14:520. doi: 10.1186/1471-2334-14-520 (PMC4261559; doi:10.1186/1471-2334-14-520)

A

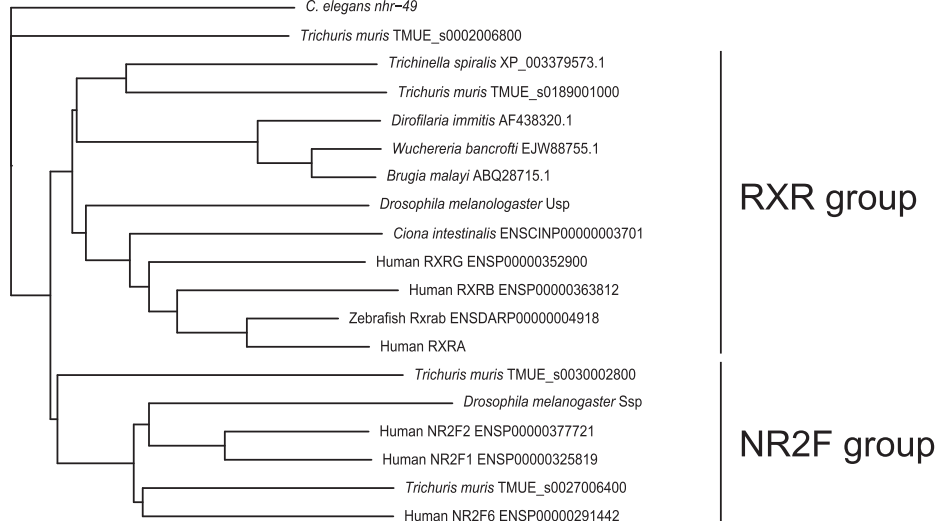

B

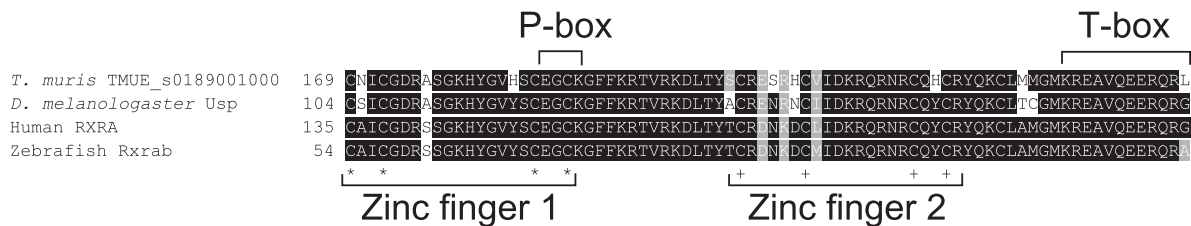

C

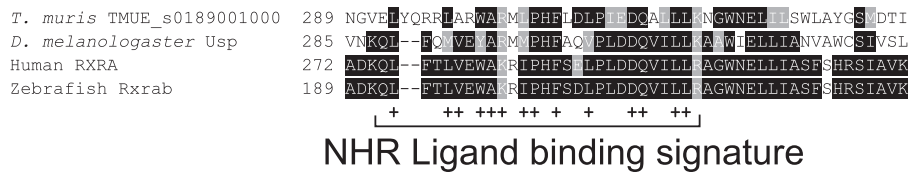

Supplement: Supplementary file 1 — Authors’ original file for figure 1 [file 12879_2014_3841_MOESM1_ESM.pdf]

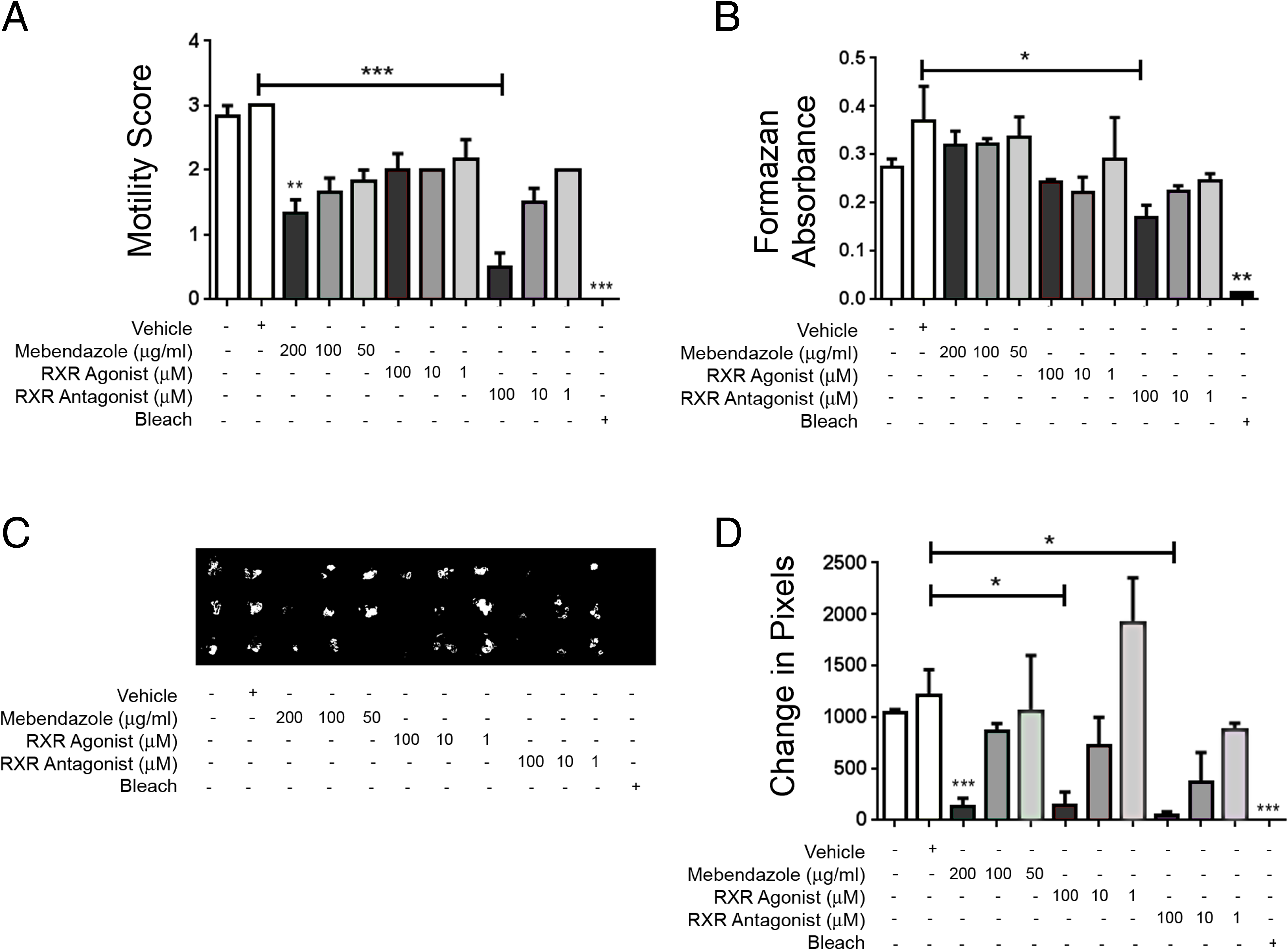

Supplement: Supplementary file 2 — Authors’ original file for figure 2 [file 12879_2014_3841_MOESM2_ESM.tiff]

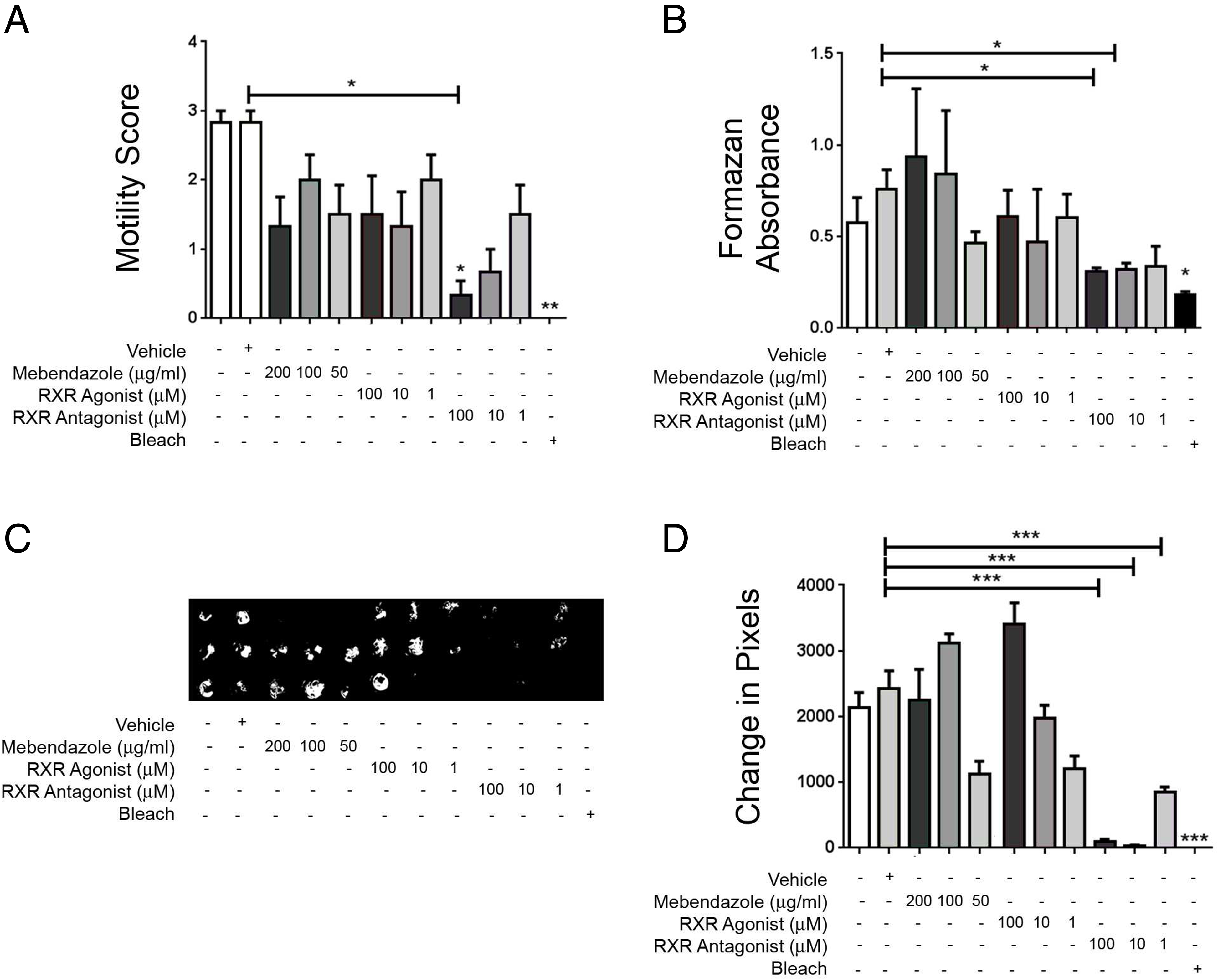

Supplement: Supplementary file 3 — Authors’ original file for figure 3 [file 12879_2014_3841_MOESM3_ESM.tiff]

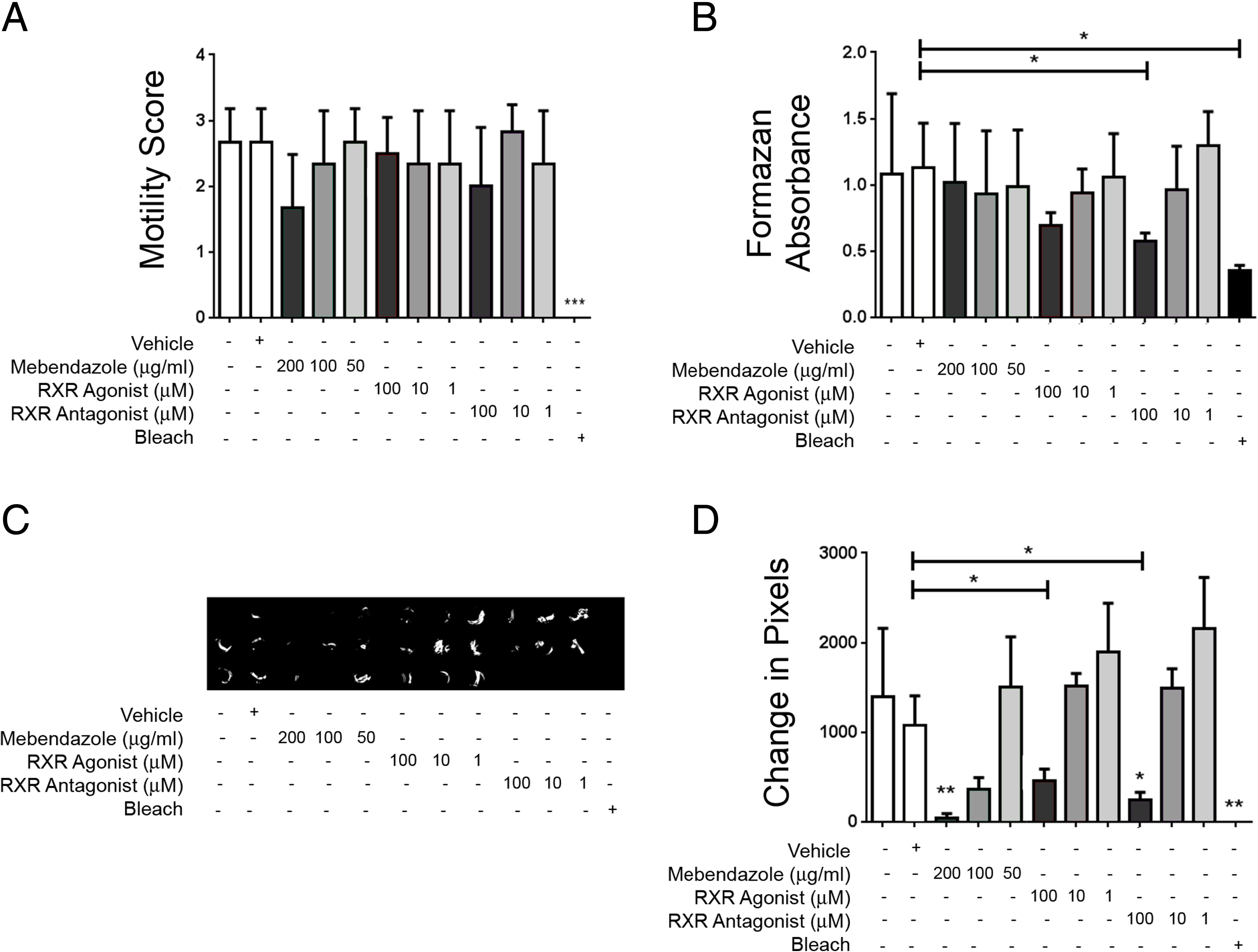

Supplement: Supplementary file 4 — Authors’ original file for figure 4 [file 12879_2014_3841_MOESM4_ESM.tiff]
